# Why AI Performs Well on Exams but Struggles With Real-World Clinical Judgment

**DOI:** 10.7759/cureus.106053

**Published:** 2026-03-29

**Authors:** Hakki Kursat Cetin

**Affiliations:** 1 Cardiac Surgery, Sisli Etfal Training and Research Hospital, Istanbul, TUR

**Keywords:** artificial intelligence, cardiac surgery, clinical judgment, medical decision-making, patient safety

## Abstract

Artificial intelligence (AI) has gained increasing attention in medicine due to its rapidly expanding capabilities in analyzing and synthesizing medical information. While AI demonstrates strong performance in structured examination settings, this success does not necessarily translate into effective real-world clinical judgment. Clinical practice, particularly in high-risk fields such as cardiac surgery, is characterized by uncertainty, incomplete information, and dynamic decision-making. This editorial examines the discrepancy between exam-based performance and clinical reasoning, emphasizing key limitations of current AI systems, including a lack of contextual awareness, susceptibility to hallucinations, limited interpretability, and the absence of accountability. It also highlights the shortcomings of exam-centered validation methods, which may overestimate clinical readiness. Ultimately, AI is best positioned as a supportive tool that augments, rather than replaces, human clinical judgment.

## Editorial

Artificial intelligence (AI) has rapidly gained prominence in medicine, largely due to its remarkable performance in standardized medical examinations. Recent studies have shown that large language models (LLMs) can answer licensing and board-style questions with high accuracy, sometimes rivaling experienced clinicians [1]. In particular, LLMs such as ChatGPT have demonstrated performance approaching or exceeding passing thresholds on standardized examinations, including the United States Medical Licensing Examination (USMLE), highlighting their ability to synthesize large bodies of medical knowledge and generate coherent clinical explanations [2]. These findings have generated considerable enthusiasm regarding the potential role of AI in clinical decision-making, medical education, and diagnostic reasoning. However, the controlled environment of standardized examinations differs fundamentally from the realities of clinical practice.

Medical exams are designed to be structured and predictable. Relevant data are provided, questions are clearly defined, and correct answers are predetermined. AI systems perform well in this setting because they excel at recognizing patterns and matching inputs to statistically likely responses [3]. In these contexts, clinical reasoning is often simplified into the retrieval of relevant information and the probabilistic selection of the most likely answer among several predefined options. Although this ability enables AI systems to achieve high scores in examination settings, it does not necessarily replicate the complexity of reasoning required in real clinical environments.

Clinical practice, by contrast, is inherently uncertain [4]. Decisions are often made with incomplete or evolving information, under time pressure, and in the presence of competing risks. In cardiac surgery, these challenges are especially pronounced. Surgeons must continuously integrate preoperative assessments, intraoperative findings, and real-time physiological changes. Patients with similar risk scores may require different operative strategies based on frailty, tissue quality, or unexpected intraoperative discoveries [5]. Furthermore, surgeons must often interpret subtle contextual cues that may not be fully captured in structured datasets, such as intraoperative tissue characteristics or rapidly evolving hemodynamic responses. These contextual elements of decision-making rely heavily on experiential knowledge and clinical intuition, which remain difficult to model within current algorithmic systems.

**Figure 1:**
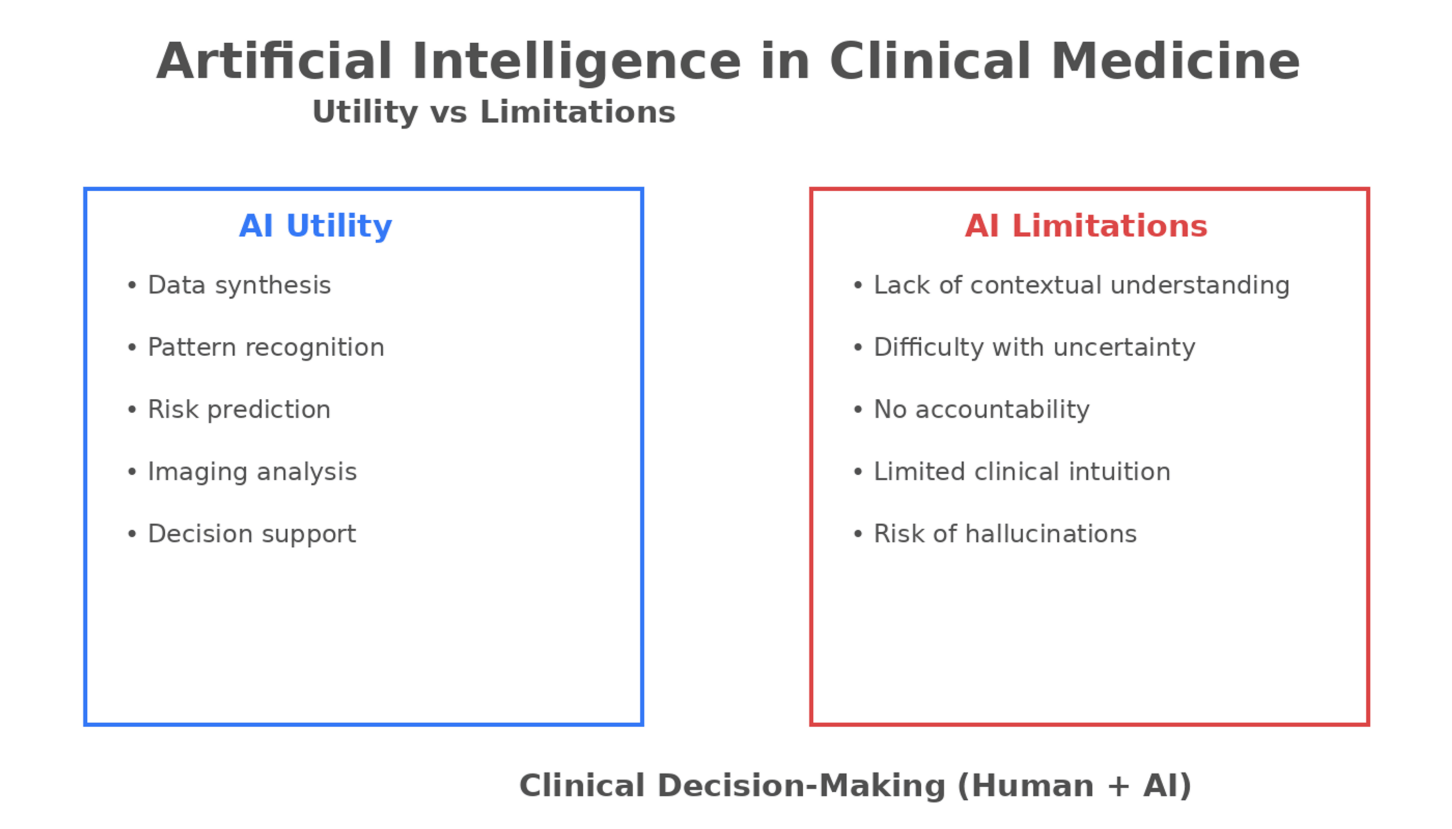
Utility and Limitations of Artificial Intelligence in Clinical Medicine Image created using Microsoft PowerPoint (Microsoft Corporation, Redmond, Washington).

The limitations of AI become most evident in situations requiring judgment rather than calculation [6]. In cardiac surgery, clinicians frequently face decisions for which no guideline provides a definitive answer, such as whether to proceed with a complex repair or modify the operative plan in response to unforeseen complications [7]. In many cases, surgical decision-making evolves dynamically throughout a procedure as new anatomical findings or physiological changes emerge. Surgeons must rapidly reassess risks, consider alternative strategies, and balance potential benefits against procedural safety. While AI can estimate probabilities, it does not assume responsibility for outcomes. Clinical judgment, however, is inseparable from accountability, a distinction that is critical in high-stakes surgical care (Figure 1).

Another important concern is the way LLMs are currently validated. Most assessments rely on exam performance, simulated scenarios, or retrospective datasets [8]. Although informative, these methods may overestimate clinical readiness. Success in examinations does not equate to managing postoperative bleeding, neurological injury, or hemodynamic instability in real time. Outcome-based, prospective validation is essential before AI-driven tools can be trusted in routine clinical workflows.

A further challenge associated with AI is the possibility of generating incorrect or misleading information, often referred to as "hallucinations" [9]. Because these systems generate responses based on statistical patterns rather than true clinical reasoning, they may produce confident but inaccurate recommendations. In high-risk specialties such as cardiac surgery, even small inaccuracies may lead to serious consequences, highlighting the need for careful human oversight and validation before clinical implementation. Rather than replacing clinicians, the most realistic near-term role for AI is likely to be augmentation of human expertise. LLMs may assist surgeons by rapidly summarizing patient data, identifying relevant risk factors, or highlighting potential diagnostic considerations [10]. However, final decision-making must remain with experienced clinicians who can integrate contextual factors, ethical considerations, and patient-specific nuances that are difficult for current algorithms to interpret. For example, AI systems may generate plausible but incorrect treatment recommendations or cite non-existent clinical guidelines, which in high-risk settings such as cardiac surgery could lead to inappropriate decision-making if not carefully verified.

Another challenge associated with LLMs in medicine is the issue of transparency and interpretability [11]. Many machine learning models, particularly LLMs and deep neural networks, function as "black box" systems in which the reasoning behind a given output is not easily interpretable by clinicians [12]. In surgical decision-making, where understanding the rationale behind a recommendation is essential for patient safety and professional accountability, limited interpretability may reduce clinician trust and hinder adoption. For high-risk specialties such as cardiac surgery, decision-support systems must not only provide accurate predictions but also allow clinicians to understand the factors influencing those predictions. Just as a surgeon would not rely solely on a single X-ray to diagnose a complex condition, so too should statistical results be considered alongside clinical expertise and a holistic understanding of the data. If this requires collaboration with AI, it can serve humanity and the medical community to a large extent [13].

AI undoubtedly has a valuable role as a decision-support tool. In cardiac surgery, it can assist with risk stratification, imaging interpretation, and data synthesis. Machine learning algorithms may help clinicians identify complex patterns in large datasets and support more informed perioperative planning. However, it should complement, not replace, human judgment. Ultimately, the most effective future model will likely involve collaboration between AI systems and experienced clinicians, combining computational power with contextual clinical understanding. Until AI can reliably navigate uncertainty and bear clinical responsibility, real-world decision-making will remain fundamentally human. Future research should focus on prospective, real-world validation and the development of explainable, context-aware AI systems that can safely integrate into clinical workflows.

## References

[1] Patel S, Ngo V, Wilhelmi B (2025). Evaluating large language models on American Board of Anesthesiology-style anesthesiology questions: accuracy, domain consistency, and clinical implications. J Cardiothorac Vasc Anesth.

[2] Kung TH, Cheatham M, Medenilla A (2023). Performance of ChatGPT on USMLE: potential for AI-assisted medical education using large language models. PLOS Digit Health.

[3] White A, Ugochukwu N, Greene J (2025). Implications for large language models and medical education in cardiac surgery. Can J Cardiol.

[4] Patel B, Gheihman G, Katz JT, Begin AS, Solomon SR (2024). Navigating uncertainty in clinical practice: a structured approach. J Gen Intern Med.

[5] Dong L, Wen F, Qin LM (2026). Associations between preoperative frailty and major postoperative complications in older surgical patients. J Clin Anesth.

[6] Parycek P, Schmid V, Novak AS (2023). Artificial intelligence (AI) and automation in administrative procedures: potentials, limitations, and framework conditions. J Knowl Econ.

[7] Dennison Himmelfarb CR, Beckie TM, Allen LA (2023). Shared decision-making and cardiovascular health: a scientific statement from the American Heart Association. Circulation.

[8] Scarfe P, Watcham K, Clarke A, Roesch E (2024). A real-world test of artificial intelligence infiltration of a university examinations system: a "Turing Test" case study. PLoS One.

[9] Neo JR, Ser JS, Tay SS (2024). Use of large language model-based chatbots in managing the rehabilitation concerns and education needs of outpatient stroke survivors and caregivers. Front Digit Health.

[10] Loftus TJ, Tighe PJ, Filiberto AC (2020). Artificial intelligence and surgical decision-making. JAMA Surg.

[11] Frasca M, La Torre D, Pravettoni G (2024). Explainable and interpretable artificial intelligence in medicine: a systematic bibliometric review. Discov Artif Intell 4.

[12] Hassija V, Chamola V, Mahapatra A (2024). Interpreting black-box models: a review on explainable artificial intelligence. Cogn Comput.

[13] Bhende VV, Sharma TS, Krishnakumar M (2024). Statistics in the operating room: a cardiovascular surgeon’s guide to numbers that matter. Cureus.

